# Malignancy and Inflammatory Bowel Disease (IBD): Incidence and Prevalence of Malignancy in Correlation to IBD Therapy and Disease Activity—A Retrospective Cohort Analysis over 5 Years

**DOI:** 10.3390/biomedicines13061395

**Published:** 2025-06-06

**Authors:** Agnieszka Jowita Kafel, Anna Muzalyova, Elisabeth Schnoy

**Affiliations:** Internal Medicine III, University Hospital Augsburg, 86156 Augsburg, Germany; agnieszkajowita.kafel@uk-augsburg.de (A.J.K.); anna.muzalyova@uk-augsburg.de (A.M.)

**Keywords:** inflammatory bowel disease, cancer, Crohn’s disease, ulcerative colitis: inflammatory bowel disease unclassified, biologics, immunosuppressive drug, Germany

## Abstract

**Background/Objectives**: Patients with inflammatory bowel disease (IBD) are at an increased risk of various cancers; such as colorectal cancer; skin cancer; bile duct cancer; or lymphoma; with IBD itself not being the sole cause. Inappropriate or ineffective IBD therapy with a continuous inflammatory burden within the gut leads to an increased risk of malignancy. Our study aimed to investigate the risk of malignancy in our patient cohort; focusing on concomitant therapy; disease duration; and inflammatory burden. **Methods**: A total of 333 consecutive adult patients with IBD (Crohn’s disease; ulcerative colitis; and IBD unclassified) were included in this study. Data from patients were collected retrospectively using patient charts. The patients were treated in the gastroenterological outpatient clinic of the University Hospital of Augsburg; Germany; between 1 January 2014 and 31 December 2018. **Results**: The study group included 333 patients; 32 (9.61%) of whom suffered from malignancy (any form). Men (*n* = 21; 65.62%) tended to develop malignancy more often than women (*n* = 11; 34.38%, *p* = 0.051). It was also observed that the probability of developing cancer was 2.40 times higher in male patients than in female patients in our cohort. However, this trend was non-significant (HR = 2.412; *p* = 0.075). Furthermore; the probability of developing cancer increased with the increasing age at the time of the first diagnosis of IBD (HR = 1.088; *p* < 0.025). A total of 20 patients (6.00%) received their cancer diagnosis after being diagnosed with IBD. The majority of those patients had skin (*n* = 6; 30.00%) or colon cancer (*n* = 5; 25.00%). Other diseases such as CML; NHL; HL; HCC; liver sarcoma; prostate cancer; breast cancer; seminoma; thyroid cancer (a second cancer in one of the patients); or CUP syndrome/lung cancer were diagnosed in single patients. Patients with IBD and colon cancer (*n* = 5; 25.00%) shared some of the known risk factors for tumour development; such as a long-lasting IBD (*n* = 5; 100.00%), diagnosis at a young age (under 30; *n* = 3; 60.00%), and the coexistence of PSC (*n* = 1; 20.00%). The cancer prevalence rate was relatively low in our cohort despite the use of diverse biologics and immunosuppressive drugs. Faecal calprotectin was confirmed as a relevant tool for inflammation monitoring in this cohort. **Conclusions**: In our study cohort; we could show a low prevalence rate of malignancy in IBD. There were more malignancies in men and in patients who were diagnosed with IBD at later ages. It can be observed that the prevalence rate of cancer was relatively low despite the use of diverse biologics and immunosuppressive drugs; which is the major conclusion of this study. Additionally; the known correlation between elevated levels of faecal calprotectin and gut inflammation was confirmed through our statistical analysis. The use of calprotectin as a non-invasive screening tool for gut inflammation is advised.

## 1. Introduction

Inflammatory bowel diseases (IBDs) include Crohn’s disease (CD), ulcerative colitis (UC), and IBD unclassified (IBDU). Chronic inflammation is most commonly found in the gastrointestinal tract. However, extraintestinal manifestations, such as skin (e.g., pyoderma gangrenosum and erythema nodosum), eyes (e.g., iritis and uveitis), bile ducts (primary biliary cholangitis (PBC) and primary sclerosing cholangitis (PSC)), or joints, can also be affected. Pathological, continuous, chronic inflammation and thereby high disease activity can increase the probability of cancer development without adequate treatment over time [1].

Colon cancer occurs more often in patients with long-lasting IBD than in the rest of the population without IBD. Patients who manifest IBD at a younger age are twice as likely to develop colon cancer as those who experience IBD at later stages of life [2]. It is imperative to have regular surveillance, such as an endoscopy.

The latest guidelines of the German Society for Digestive and Metabolic Diseases (Deutsche Gesellschaft für Gastroenterologie, Verdauungs- und Stoffwechselkrankheiten (DGVS)), updated in 2020, 2024, and 2025, recommend that the first screening colonoscopy in patients with UC should be performed 6–8 years after initial diagnosis. The risk of developing cancer (classified into three categories: low, intermediate, and high risk) should be a decisive factor in determining the interval for surveillance for each patient individually [3]. The same prophylaxis should be advised in patients with CD.

The ECCO (European Crohn’s and Colitis Organisation) guidelines recommend a screening colonoscopy in patients with UC from the eighth year of disease onset, depending on its activity. Patients with a high risk of cancer, e.g., with highly active disease or coexisting PSC, should undergo colonoscopy yearly, regardless of the activity or expansion of the disease (as also recommended in the German guidelines). For patients with an intermediate risk of cancer development, colonoscopies, e.g., with ongoing mild inflammation, should be performed every 2 or 3 years, and for those with a low risk, every 4 years [4,5].

It is well known that patients with IBD can also develop other malignancies such as skin cancer, bile duct cancer, or lymphoma. Moreover, patients with CD have an increased risk of gastric or lung cancer. Leukaemia can be more often detected in patients with UC [4,5].

IBD treatment in the acute phase is primarily based on prednisolone and aminosalicylates (for UC). In the case of a refractory disease, drugs from other groups, such as immunomodulators (e.g., azathioprine, mercaptopurine, and methotrexate) [6] or biologics (e.g., infliximab, adalimumab, and ustekinumab) should be considered. It is essential that this treatment leads to disease remission and stops the inflammatory activity [3]. It has been recently confirmed that full mucosal healing is accompanied by a better IBD long-term prognosis [2,7].

IBD therapy itself can also influence the development of cancer. The use of thiopurines can lead to nonmelanoma skin cancer without proper prophylaxis, including regular skin checks every year. A therapy with biologics (TNF-inhibitors) can increase the risk of melanoma [4,8,9]. An immunomodulatory therapy can also increase the risk of pathological Papanicolaou smear [8] and cervix dysplasia. Therefore, regular annual gynaecological check-ups and HPV vaccination are also recommended for young female patients [8].

This retrospective study was focused on a single-centre group of patients with IBD and their risk of cancer in a real-world setting. Our aim was to answer the question of whether advanced IBD therapies and IBD activity had an influence on the development of cancer in this real-world cohort.

## 2. Materials and Methods

### 2.1. Study Population

This was a monocentric, retrospective, descriptive study performed at the University Hospital Augsburg, Germany, in accordance with the Declaration of Helsinki and Good Clinical Practice (ethics committee vote No. 23-3209-104, University of Regensburg, Germany). All of the adult patients (*n* = 333) with IBD who were treated in the gastroenterological outpatient clinic of the University Hospital Augsburg between 1 January 2014 and 31 December 2018 were screened and included in the study.

The following data were collected: date of birth, gender, date of death, date of the IBD diagnosis, IBD subtype, and medical treatment. In the cancer patients with IBD, the following data were also assembled: date of cancer diagnosis, type of cancer, stage, and cancer therapy (operation, radiotherapy, chemotherapy, immune therapy, and antibody therapy). In the patients who were first diagnosed with IBD and then with cancer, data were also collected on all IBD therapies since the IBD diagnosis, including mean endoscopic activity (colonoscopy) before, at the time of, and after the cancer diagnosis; all calprotectin levels in stool samples were noted.

IBD activity was determined based on the results from endoscopic examinations (colonoscopies) conducted in the hospital and the levels of calprotectin in the stool. The amount of calprotectin and the results of endoscopic tests were too low for the calculations; therefore, some previous results (prior to 2014) were also included. Due to some changes in the laboratory units, 600 µg/g was used as the maximum level of calprotectin. The normal level was considered less than 50 µg/g.

For this study, the descriptive “activity classification” was used, dependent on the macroscopic inflammatory activity in the colon tissue: no activity (0), low (1), mild (2), significant (3), and pronounced [4] (Table 1).

Only endoscopic results from patients first diagnosed with IBD and then with cancer were included. Patients with UC also received at least one sigmoidoscopy. Those results were not included in the calculations.

All the above-mentioned patient data were obtained through the ORBIS^®^ electronic documentation system (version 08043501.04100.DACHL; DH Healthcare GmbH/company with limited liability) or from external sources (contact with other hospitals or patients´ general practitioners). In two patients with IBD and cancer, the date of IBD diagnosis was not known, and in three patients, the date of cancer was not known; they were therefore not included in the calculations, but they were mentioned in this study.

In two patients, who died between 2014 and 2018, calculations included the age of death (as the patient´s age). The disease duration was calculated until the year of death.

In the case of four patients with two different cancers, only the earlier date of the cancer diagnosis was included in the calculations.

### 2.2. Statistical Analysis

The data were then collected and retrospectively analysed descriptively in Microsoft Excel 2007.

Categorical variables were reported in absolute numbers and percentages. The percentage numbers were rounded to the second decimal place.

Continuous variables were presented as mean values with standard deviation (SD) and/or median and interquartile range (IQR), as appropriate for the data. Additionally, ranges consisting of minimal and maximal values were calculated.

The chi-squared test was used to calculate the association between categorical variables. The Kendall’s Tau test was used to evaluate the correlation between ordinal-scaled variables. Differences between mean values were calculated using the Mann–Whitney U test or the Kruskal–Wallis test, depending on the number of independent groups to be compared. The risk factors for the development of tumours were analysed with the help of Cox regression.

The significance level was set at α = 0.05. Graphics were created in Microsoft^®^ Excel and Word 2007. Statistical analysis was performed using SPSS 28.0 (Statistical Package for Social Sciences). The reference list was partially created using Citavi Free 6.8 reference management software.

## 3. Results

### 3.1. Patient Cohort

**Figure 1:**
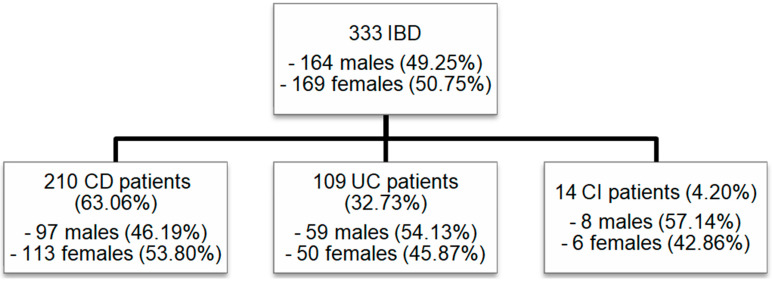
Patient cohort: Numbers of male and female patients included in our study with IBD (CD, UC, and IBDU). IBD: inflammatory bowel disease; CD: Crohn’s disease; UC: ulcerative colitis; IBDU: IBD unclassified.

The group consisted of 333 patients, including 164 males (49.25%) and 169 females (50.75%). A total of 210 (63.06%) patients suffered from CD (97 males, 46.19%, and 113 females, 53.80%; *p* = 0.145). UC was diagnosed in 109 (32.73%) patients (59 males, 54.13%, and 50 females, 45.87%; *p* = 0.214). A total of 14 (4.20%) patients (8 males, 57.14%, and 6 females, 42.86%; *p* = 0.546) had IBDU (Figure 1).

The mean age of patients in the entire study group was 46.83 (SD = 15.64; median = 47; IQR = [58–33]) years. There was a minor non-significa9nt difference between male (46.79 (SD = 16.43) years) and female patients (46.86 (SD = 14.53) years; *p* = 0.777). The oldest patients in the cohort were females with IBDU (52.67 (SD = 16.50) years). Males with IBDU were the youngest (40.13 (SD = 14.29) years; Table S1).

Patients were diagnosed with IBD at the age of 31.55 (SD = 14.15; median = 28; IQR = [39–21]). Male patients were 32.13 (SD = 15.08) years old, and female patients 30.98 (SD = 12.98) years old. Patients with CD were diagnosed at the age of 31.01 (SD = 13.70) years. UC was detected at the age of 32.74 (SD = 14.61) years. The average age of the IBD diagnosis in the patients with IBDU was 30.43 (SD = 14.62) years (Table S1).

The disease duration in IBD patients in the whole cohort at the time of the study was diverging, ranging from 1 to 47 years, with a mean of 15.13 (SD = 10.82; median = 13; IQR = [22–6]) years. IBD in the group of UC patients lasted for 12.58 (SD = 9.48) years; in contrast, in the group of patients with IBDU, it lasted for 15.07 (SD = 8.70; range = 4–33) years. Patients with CD showed the longest disease duration in comparison to the study subgroups mentioned above (16.45; SD = 11.45; range = 1–44; *p* = 0.333) (Table S1).

### 3.2. IBD and Cancer

Cancer was diagnosed in 32 of the 333 (9.61%) patients (21 males, 65.62%; 11 females, 34.38%), and there was a tendency that male patients suffered more often from cancer than female patients (*p* = 0.051).

It was also detected that the probability of cancer development was 2.40 times higher in male patients than in females in our cohort. However, this trend was non-significant (HR = 2.412; *p* = 0.075; Table 2). The statistical analysis also showed that the probability of developing cancer increased with increasing age at the first diagnosis of IBD (HR = 1.088; *p* < 0.025; Table 2).

There were three male patients (2 CD, 1 UC) and one female patient with CD who suffered from two different malignant diseases.

A total of 20 patients (62.50%) with cancer had CD (11 males (34.38%); 9 females (28.13%)). A total of 11 (34.38%) cancer patients suffered from UC (9 males (28.13%); 2 females (6.25%)), and 1 (3.13%) male had IBDU.

The mean age of cancer patients with IBD at the time of the study was 57.88 (SD = 14.98; median = 60; IQR = [68.75–47.25]) years. The oldest patients with UC were 63.56 (SD = 19.61) years (male) and 63.50 (SD = 19.09) years (female); *p* = 0.639. Cancer patients were diagnosed with IBD at the age of 40 (SD = 17.69; Tables S2 and S3).

The duration of IBD disease in cancer patients was 1–47 years, with a mean disease duration of 17.03 (SD = 11.71, median = 14.00) years. In the group of male patients, IBD disease duration was longer (17.35 (SD = 11.70); range = 2–47 years; median = 14.00; IQR = [18.00–11.00]; *p* = 0.588) than in the group of female patients (16.40 (SD = 14.34); range = 1–42 years; median = 13.00; IQR = [17.75–11]). The longest IBD duration was detected in male patients with CD, ranging from 11 to 44 years (19.40 (SD = 10.38); median = 15.50; IQR = [22–11]). In patients with UC and cancer, the disease duration for UC was 15.40 years on average (SD = 12.20; range = 2–47 years; median: 14.00; IQR = [16.5–8]). The only cancer patient with IBDU had suffered from IBDU for 13 years (Table S4). No significant differences were detected in disease duration across all study subgroups (*p* = 0.154). Cancer was diagnosed, on average, at the age of 48.34 (SD = 14.56; median = 48.00; IQR = [59–36]; Table S2).

**Figure 2:**
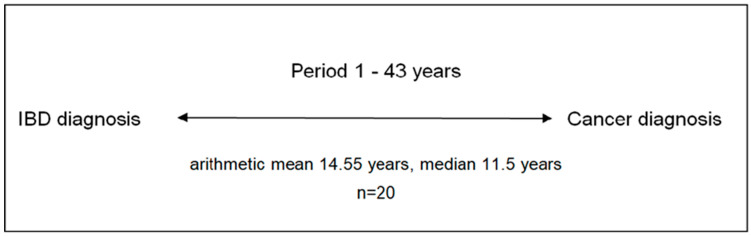
Period between IBD and cancer diagnosis in 20 patients with IBD and cancer. IBD: inflammatory bowel disease.

A total of 20 patients were first diagnosed with IBD and then 14.55 years (median = 11.50 years; range = 1–43 years; IQR = [17.5–6.5]) later with cancer (Figure 2).

There was no association between diverse IBD subtypes (CD, UC, or IBDU) and cancer development (*p* = 0.938).

### 3.3. Cancer and IBD Therapy

A total of 32 patients in this study were cancer patients; this group was divided into 12 main groups: skin cancer (10, 31.25%) (squamous cell carcinoma (SCC), melanoma, basal-cell carcinoma (BCC), or highly differentiated verrucous carcinoma/Buschke–Löwenstein tumour (BLT)), gastrointestinal cancer (9, 28.12%) (small intestine cancer, colon cancer, and neuroendocrine tumour (NET)), haematologic diseases (5, 15.62%) (acute myeloid leukaemia (AML), chronic myeloid leukaemia (CML), non-Hodgkin lymphoma (NHL) or Hodgkin lymphoma (HL)), liver cancer (2, 6.25%) (hepatocellular carcinoma (HCC) and liver sarcoma), prostate cancer (2, 6.25%), breast cancer (1, 3.12%), renal cell carcinoma (1, 3.12%), seminoma (1, 3.12%), thyroid cancer (1, 3.12%), CUP syndrome (cancer of unknown primary) (1, 3.12%), and/or otorhinolaryngological tumours (2, 6.25%) (tongue and tonsil cancer) (Table S4).

A total of 20 patients first diagnosed with IBD and later with cancer were additionally marked in the table. The IBD therapies of those 20 patients included immunomodulators (azathioprine, mercaptopurine, cyclosporin A, and methotrexate) and biologics (infliximab, adalimumab, golimumab, ustekinumab, and vedolizumab) (Table S5). Certolizumab was used in two patients as a therapy for concomitant rheumatoid arthritis. It is not approved as an IBD therapy in Germany.

**Figure 3:**
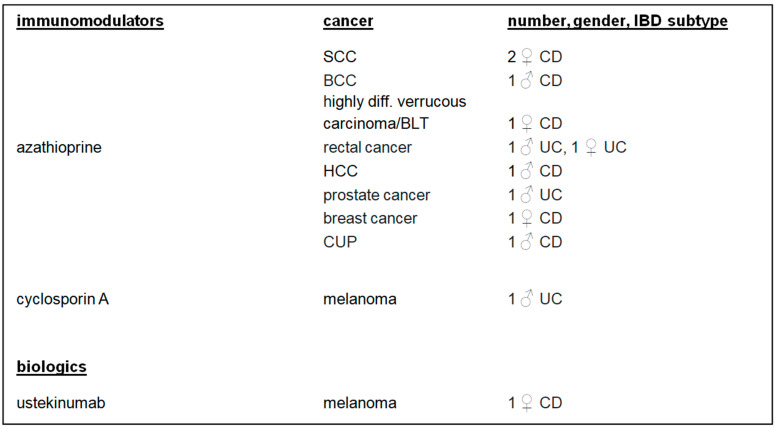
IBD monotherapy, cancer, number, gender, and IBD subtypes. IBD: inflammatory bowel disease; SCC: squamous cell carcinoma; BCC: basal-cell carcinoma; HCC: hepatocellular carcinoma; cancer of unknown primary.

**Figure 4:**
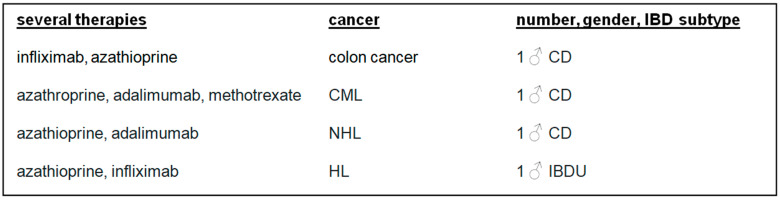
Several IBD therapies, cancer, number, gender, and IBD subtypes. CML: chronic myeloid leukaemia; NHL: non-Hodgkin lymphoma; HL: Hodgkin lymphoma, IBD: inflammatory bowel disease.

There were 10 (50.00%) patients who developed cancer after or during therapy with azathioprine. Melanoma was diagnosed in two (10.00%) patients treated with cyclosporin A or ustekinumab. Four (20.00%) patients developed cancer after IBD combination therapies (Figure 3 and Figure 4).

### 3.4. IBD Activity

Disease activity was analysed in detail in patients with cancer and IBD using the endoscopy (colonoscopy) and stool test (faecal calprotectin) results. In the course of study, 171 colonoscopies were conducted.

A total of 13 (65.00%) out of 20 patients were investigated in our department before cancer diagnosis. Those patients had most frequently mild (5, 38.46%) and low (4, 30.77%) macroscopic activity in the colon. Significant and pronounced activity was observed in each case in only one patient (7.69%, 7.69%). Two (15.38%) patients had no macroscopic activity in the colon.

After a cancer diagnosis, 13 (65.00%) patients had follow-up colonoscopies within our department. Four patients (30.77%) in this subgroup did not exhibit any macroscopic signs of inflammation in the colon. Mild and significant activity was found in three patients in each group (23.08%, 23.08%). Pronounced activity was detected in two patients, and low activity was found in one patient (7.69%).

**Figure 5:**
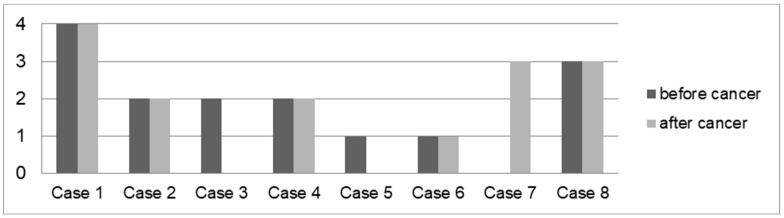
Average macroscopic IBD activity in the colon before and after cancer diagnosis in eight patients with IBD and cancer. Cases: 1: IC, HL Hodgkin lymphoma, 2: UC, thyroid and rectal cancer, 3: CD, melanoma, 4: UC, prostate cancer, 5: UC, rectal cancer, 6: UC, sarcoma, 7: CD, NET of appendix (neuroendocrine tumour), 8: CD, CUP syndrome (cancer of unknown primary).

A total of 8 out of 20 (40.00%) patients had colonoscopies before and after cancer diagnosis (Figure 5). Five (62.50%) patients exhibited the same level of activity in the colon (low: 1 case; mild: 2 cases; significant: 1 case; pronounced: 1 case) before and after their cancer diagnosis. Two (25.00%) patients had low (1 case; 12.50%) or mild (1 case, 12.50%) activity and no activity after cancer diagnosis. One (12.50%) patient did not have any macroscopic inflammatory activity in the colon before the cancer diagnosis. After the cancer diagnosis, significant inflammation was detected.

**Figure 6:**
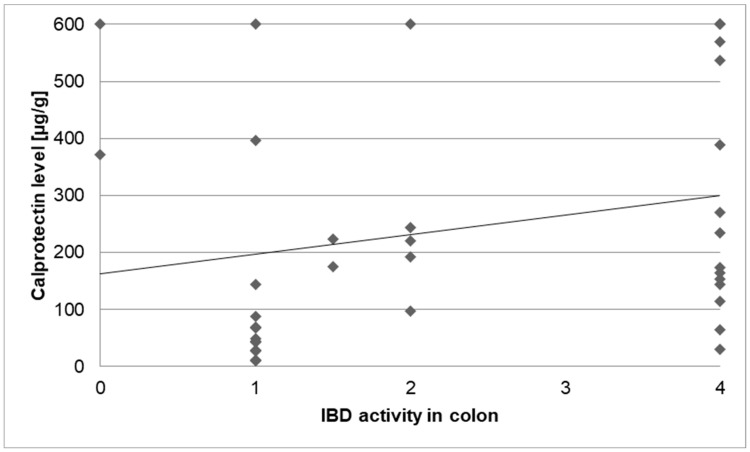
Correlation of IBD activity in the colon (0–4) and calprotectin levels.

To define IBD activity in the colon before cancer diagnosis, calprotectin levels and the macroscopic activity in the colon (as defined by the numbers in the “activity classification” mentioned above) were paired, depending on the date of the test and examination. A statistically significant correlation was found between endoscopic activity and calprotectin level (τ = 0:257; *p* = 0.046; Figure 6).

## 4. Discussion

### 4.1. Patients with IBD and Cancer

Our study investigated patients with IBD and the risk of malignancy with a focus on concomitant IBD therapy and inflammatory burden.

A total of 333 patients were included in our study; most patients suffered from CD, with a higher incidence in women than in men. In the case of UC, it is more often found in men than in women (8). IBD subtypes were found equally in both sexes.

On average, IBD was diagnosed at the age of 31.55, which corresponds to the peak age for the onset of IBD in younger age groups. Although IBD can be diagnosed at any age, it is most frequently found (60-85%) under the age of 40 [6].

Only 32 patients suffered from cancer in this study. In those patients, IBD was diagnosed, on average, 8.45 years later than in the non-oncological patients. We demonstrated that the probability of developing cancer increased significantly with increasing age at the time of the first diagnosis of IBD. For the risk of colon cancer in IBD patients, the opposite was observed. Patients with an onset of IBD at a young age have a twofold increased risk of colon cancer compared to patients who developed IBD in adulthood [2]. Tight surveillance strategies for colorectal cancer screening via colonoscopy (e.g., a first surveillance colonoscopy around 6-8 years after the onset of IBD, followed by subsequent colonoscopies in accordance with individual risk factors for patients) are recommended by national and international guidelines [4,5]. It is known that patients with IBD have a higher risk of colorectal cancer than healthy controls [10,11]. Long-lasting UC increases the risk of colon cancer [12] by 1.60% after 10 years, 8.30% after 20 years, and by 18.40% after 30 years [13,14]. In addition, there are some other aspects, such as early onset of IBD, including IBD location, high disease activity, coexistent PSC, a positive family history regarding colon cancer, and IBD manifestation, that contribute to this risk [4,15,16]. High disease activity (extended and left-sided) in UC increases the risk of cancer development by up to two–three times [5]. Therapy with 5-aminosalicylates (e.g., mesalazine) for UC may be protective [15,17], but only when it is regular and long-term [18,19].

In our cohort, most of our patients with cancer were males, and it was also observed that the probability of developing cancer was 2.4 times higher in male patients than in females. Our data are in accordance with the results of a large US cohort, where men with IBD showed a higher risk of cancer development in most shared anatomic regions than women [20]. However, the statistical analysis of our data was limited due to the retrospective design and the low number of patients with cancer. This should be taken into account when interpreting the data.

### 4.2. Cancer Subtypes in Patients with IBD

In our study, there were eight patients diagnosed with colon cancer. In five patients, the cancer was detected after the onset of IBD. Three of those patients suffered from CD and two from UC. Unfortunately, the number of patients was not high enough for a statistical analysis. IBD disease duration until a cancer diagnosis in those five patients was from 12 to 41 years, such that, in every case, long-lasting IBD was an underlying risk factor for cancer development, as described in the literature [5,15,16]. Three out of five cases were diagnosed at a young age, which was below 30 years [17,21] and 28 years of age; this is another well-known risk factor for cancer development [5,15,16]. The two remaining patients were diagnosed with IBD after the age of 30 (36 and 42 years of age). Therefore, our data also show that long-lasting diseases are risk factors for the development of subsequent cancers.

One female patient with UC and rectal cancer also suffered from PSC. The risk of cancer in patients with IBD and PSC is three times higher than in patients with IBD but without PSC, and especially when they suffer from UC [21]. In such cases, colon cancer can be detected more often in the right colon [21]. This is why disease activity must be controlled regularly through endoscopic surveillance strategies. The patient described was diagnosed with rectal cancer 12 years after the first IBD diagnosis. The patient was given intermittent mesalazine for 5 years and azathioprine for 3 years. Unfortunately, it was not evident if she also received ursodeoxycholic acid (UDCA) as a standard therapy for PSC. In patients with PSC, yearly surveillance colonoscopies should be performed.

Small intestine cancer is a rare cancer which can occur more often in patients with CD and the involvement of the small intestine [21,22,23,24]. The prevalence is about 1.6%, and the prognosis is often rather bad [24]. In our study, a male patient with CD and NET was identified in the terminal ileum. There are Danish and Swedish studies showing that the risk of cancer development of all histological subtypes (adenocarcinoma, neuroendocrine tumour, and sarcoma) of small intestine cancer is increased [23] in PBD. In our study, the cancer was diagnosed in the same year as IBD was first detected; this is the reason why these might have been two independent factors in this special patient.

To sum up, besides many other cancer types, patients with CD also have an increased risk of stomach cancer [22]. This study did not cover such a case.

### 4.3. Second Cancer in Patients with IBD

There were four patients with two different cancer diseases diagnosed. It is well known that the risk of developing a second cancer in a patient with IBD and an already diagnosed cancer disease is twice as high as in IBD patients without cancer [22,25]. The second cancer is usually detected in male patients with IBD who are more than 50 years old [25]. In our study, two patients exhibited this phenomenon. So, patients with a history of cancer need to have tight surveillance strategies due to the risk of a second cancer in the follow-up.

So far, extraintestinal cancer has been discussed only in a few big population-based studies focused on cancer in IBD patients [21], although it is known that patients with IBD are at a higher risk of developing extraintestinal cancer. A Danish study investigating a total of 50 studies showed that patients with IBD have a higher risk of developing skin and hepatobiliary cancer [10]. The group of patients with CD also showed a tendency for haematologic diseases and lung cancer. Moreover, a higher risk of urothelial carcinoma in patients with CD and a higher risk of liver/bile duct cancer in patients with UC were also mentioned in other studies [22,25]. In our study, there were single patients with diagnosed CML, NHL, HL, HCC or CUP syndrome/lung cancer.

It is a fact that patients with IBD are at a risk of non-melanoma (SCC, BCC) and melanoma (more commonly associated with the IBD therapy) [22]. Moreover, often-prescribed immunomodulators in patients with IBD, such as the thiopurines (azathioprine, 6-mercaptopurine), can increase the risk of the development of non-melanoma skin cancer and lymphoma [26,27,28]. The optimal duration of therapy, which can either increase or decrease this risk, is, unfortunately, unknown [27]. However, the risk of side effects increases over time. The probability of lymphoma development can even be four times higher compared to a healthy population [28,29]. A cohort from the CESAME study, which included more than 20,000 patients with IBD, even mentioned a fivefold higher risk of lymphoma under therapy with thiopurines [17]. Although thiopurines can also decrease the risk of cancer, as confirmed in a Chinese study, those medications can protect against the development of colon cancer [14]. It is also a fact that another specific IBD therapy, anti-TNF antibodies, can increase the risk of skin cancer, such as melanoma [26]. Our study included six patients who were first diagnosed with IBD and then with skin cancer. Two female patients with CD had SCC after and during the therapy with azathioprine. A male patient with CD was undergoing therapy with azathioprine when BCC was diagnosed. The disease itself and the therapy with azathioprine might be interpreted as risk factors for SCC/BCC development in those patients. Melanoma was detected in two patients: a male patient with UC was on therapy with cyclosporine A, and a female patient with CD was receiving biological therapy (ustekinumab) when skin cancer was diagnosed. However, the number of patients in our cohort is too small to demonstrate a significant trend or correlation with any of these therapies.

To prevent the development of skin cancer under thiopurines or biologics, patients’ skin should be monitored (through skin cancer screening) once a year [30]. Moreover, protective measures from solar ultraviolet radiation should be advised [31].

### 4.4. Cancer in IBD and Concomitant Medication

In recent years, there have been studies focusing on cancer development as a side effect during therapy with biologics. An overview of 28 different studies revealed no correlation between cancer and treatment with anti-TNF antibodies [29]. However, there was a study which showed an increased risk of lymphoma. This risk increased even more when combination therapy was used, as mentioned below [29]. Two of our study patients with lymphoma (NHL, HL) received combination therapies. So, patients with combination therapies should be closely monitored for the development of lymphoma. A combination therapy is often prescribed as IBD treatment. However, it cannot be overlooked that a simultaneous combined therapy with anti-TNF antibodies and thiopurines may increase the risk of lymphoma compared to monotherapy with thiopurines [27]. It was also reported that the combination of thiopurines and infliximab increases the risk of hepatosplenic T-cell lymphoma [26]. It is even more risky for young men without EBV-titre when given azathioprine or mercaptopurine for IBD treatment [25].

Infliximab, which has been used the longest, can also be prescribed in the treatment of rheumatoid arthritis and IBD. A randomised study on rheumatological patients receiving infliximab showed a rare development of malignant diseases with no statistical significance [32]. Another prospective cohort study revealed that rheumatological patients, who received anti-TNF treatment, suffered more often from haematologic diseases than those who received, e.g., methotrexate [32]. In 2017 there was a prospective study from Hyams and colleagues on 5766 patients, which revealed that infliximab does not increase the risk of cancer development in paediatric patients with IBD [33]. The data mentioned concerning cancer development during therapy with biologics are being disputed, so it is crucial to further investigate this issue.

Four of our patients received several IBD drugs before cancer was diagnosed, meaning that it was not possible to clearly analyse which of the medications might have influenced cancer development. Regardless of the medications used here, the cohort analysed and treated in a specialised university outpatient clinic with the need for biologic therapy due to higher inflammation burden has a higher risk of developing cancer, which might be a bias in our cohort.

As the patient subgroups with various cancer diseases in our study were too small, the statistical analysis of the correlation between IBD therapy and cancer development did not reveal any statistical endpoint. Faecal calprotectin is a reliable tool for controlling inflammatory activity in the colon [2], with a level lower than 150–200 µg/g indicating remission [34]. Our study confirmed a correlation between elevated faecal calprotectin levels and inflammation in the colon. In our cancer patients, mild activity in the colon was detected in 20 cases. A good example was a male patient with UC who was diagnosed with two different cancer diseases: thyroid and rectum cancer, with a malignant process in the gastrointestinal tract, which was detected after a long-lasting UC. The endoscopic controls, which were unfortunately irregular, showed mild macroscopic activity in the colon. No calprotectin levels were documented. As the correlation between high calprotectin levels and inflammation in colon tissue is well-established, it is advisable to utilise it as a non-invasive tool for monitoring ongoing inflammation, thereby reducing the risk of cancer development in the long term.

## 5. Conclusions

Although the question of whether the use of biologics can increase the risk of cancer development remained unanswered in our study due to a low rate of cases with cancer, it is worth noting that the cancer prevalence in our study was relatively low over a long period of time, despite the use of diverse modern biologics and immunosuppressive drugs, which is the major conclusion of this study.

In our monocentric study with more than 330 IBD patients, there was only a minority of 32 patients who developed cancer. Compared to the literature, the cancer prevalence rate was relatively low. The known risk factors for tumour development could be confirmed, such as a long-lasting IBD, diagnosis of IBD at a young age (under 30), and the coexistence of PSC. A conscientious prophylaxis in those at-risk patients is advised and has to be conducted, first and foremost, in patients with an early-onset IBD. Due to the retrospective design, underreporting might apply, and some cancer diagnoses might have been missed due to loss of follow-up. On the other hand, the clear and standardised recommendations at our university hospital, e.g., for surveillance colonoscopies or regular skin controls, might have contributed to a low cancer incidence in our cohort.

It is worth noting that IBD therapies used in the treatment of our study patients tended not to cause increased cancer development. Although the patient numbers were relatively low, no statistical significance was found; therefore, further prospective studies, such as multicentre studies, are warranted to clarify this question. Another relevant result was that male patients in our study tended to develop malignancy more often than female patients.

A statistically significant correlation between IBD activity and faecal calprotectin levels was also detected; this should still be consistently observed on a daily basis in practice to monitor disease activity, as patients with a higher inflammatory burden have a higher risk of cancer development over time.

Adequate surveillance and control of inflammation in the gut are key to reducing and better preventing malignancy in IBD patients. Individual screening programs need to be applied in accordance with the patient’s risk profile (yearly, every 2–3 years, every 4 years).

Due to the growing incidence of IBD in society, which is a well-known issue, as well as the occurrence of new therapeutic possibilities, it seems reasonable to conduct such studies again in a larger prospective patient cohort in multiple centres.

## Figures and Tables

**Table 1:** Intensity of macroscopic inflammatory activity in the colon tissue according to grade (0 = no; 1 = low; 2 = mild; 3 = significant; 4 = pronounced).

Macroscopic IBD Activity in Colon	Grade
No	0
Low	1
Mild	2
Significant	3
Pronounced	4

**Table 2:** Cox regression of risk factors for tumour development in patients with IBD. IBD: inflammatory bowel disease.

	Hazard Ratio (HR)	Confidence Interval		*p*-Value
		Lower Bound	Upper Bound	
Gender: men	2.412	0.914	6.367	0.075
Age	0.939	0.873	1.01	0.093
Age at IBD diagnosis	1.088	1.011	1.171	0.025

## Data Availability

All data generated or analysed during this study are included in this article and its Supplementary Materials. Further inquiries can be directed to the corresponding author.

## Supplementary Materials

The following supporting information can be downloaded at https://www.mdpi.com/article/10.3390/biomedicines13061395/s1. Table S1: ages (at the time of the study, at the IBD diagnosis) and IBD duration of study patients. Table S2: age (at the time of the study, at the IBD diagnosis) and IBD duration of cancer patients. Table S3: age at the diagnosis of cancer. Table S4: characteristics of cancer patients with IBD considering the main groups of cancer, sex, age at the IBD diagnosis, age at the cancer diagnosis, year and age at the death. (*1) Patient with rectal and thyroid cancer. (*2) Patient with NET in term. Patients who first had IBD and then cancer were additionally marked in the table. Table S5: characteristics of cancer patients with IBD considering the cancer diagnosis (incl. stage, the date of diagnosis), IBD, sex, year of the IBD diagnosis, cancer therapy and IBD therapy (before and after the cancer diagnosis). (*1) Patient with rectal and thyroid cancer; (*2) Patient with NET in terminal ileum and CML; (*3) Patient with tongue and tonsil cancer.

## Abbreviations

AML	acute myeloid leukaemia (AML)
BCC	basal-cell carcinoma
BLT	Buschke–Löwenstein tumour
CD	Crohn’s disease
CML	chronic myeloid leukaemia
CUP	cancer of unknown primary
DGVS	German Society for Digestive and Metabolic Diseases; Deutsche Gesellschaft für Gastroenterologie, Verdauungs- und Stoffwechselkrankheiten
ECCO	European Crohn’s and Colitis Organisation
HL	Hodgkin lymphoma
HR	hazard ratio
HCC	hepatocellular carcinoma
IBD	inflammatory bowel disease
IBDU	inflammatory bowel disease unclassified
IQR	interquartile range
NET	neuroendocrine tumour
NHL	non-Hodgkin lymphoma
PBC	primary biliary cholangitis
PSC	primary sclerosing cholangitis
SD	standard deviation
SCC	squamous cell carcinoma
UC	ulcerative colitis

## References

[1] Seyedian S.S., Nokhostin F., Malamir M.D. (2019). A review of the diagnosis, prevention, and treatment methods of inflammatory bowel disease. J. Med. Life.

[2] Olén O., Askling J., Sachs M., Frumento P., Neovius M., Smedby K., Ekbom A., Malmborg P., Ludvigsson J. (2017). Childhood onset inflammatory bowel disease and risk of cancer: A Swedish nationwide cohort study 1964–2014. BMJ.

[3] Wehkamp J., Götz M., Herrlinger K., Steurer W., Stange E.F. (2016). Inflammatory Bowel Disease. Dtsch. Arztebl. Int..

[4] Medycyna P. (2014). Interna Szczeklika: Podręcznik Chorób Wewnętrznych.

[5] Messmann H., Andus T., Baum K. (2012). Klinische Gastroenterologie: Das Buch Für Fort-Und Weiterbildung.

[6] Prelipcean C.C., Mihai C., Gogalniceanu P., Mihai B. (2013). What is the Impact of Age on Adult Patients with Inflammatory Bowel Disease?. Clujul Med..

[7] de Weerth A., Bläker M. (2016). Chronisch-Entzündliche Darmerkrankungen. Hambg. Ärzteblatt.

[8] Peppercorn M.A., Cheifetz A.S. (2019). Definitions, Epidemiology, and Risk Factors for Inflammatory Bowel Disease in Adults. https://www.uptodate.com/contents/definitions-epidemiology-and-risk-factors-for-inflammatory-bowel-disease.

[9] Friedman G., Bitton A. (2020). Overview of Hepatobiliary Disorders in Patients with Inflammatory Bowel Disease. https://www.uptodate.com/contents/overview-of-hepatobiliary-disorders-in-patients-with-inflammatory-bowel-disease.

[10] Lo B.Z.S., Zhao M., Vind I., Burisch J. (2020). The risk of extra-intestinal cancer in inflammatory bowel disease (IBD): A systematic review and meta-analysis of population-based cohort studies. J. Crohn’s Colitis.

[11] Zisman T.L., Rubin D.T. (2008). Colorectal cancer and dysplasia in inflammatory bowel disease. World J. Gastroenterol..

[12] Timeus S., Laoun R. (2020). Risk of colorectal cancer and other malignancies in association with the use of thiopurines, tumour necrosis factor antagonists or mesalazine. J. Crohn’s Colitis.

[13] Horio Y., Uchino M., Bando T., Sasaki H., Goto Y., Kuwahara R., Minagawa T., Takesue Y., Ikeuchi H. (2020). Incidence, Risk Factors and Outcomes of Cancer of the Anal Transitional Zone in Patients with Ulcerative Colitis. J. Crohn’s Colitis.

[14] Zhu Z., Mei Z., Guo Y., Wang G., Wu T., Cui X., Huang Z., Zhu Y., Wen D., Song J. (2018). Reduced Risk of Inflammatory Bowel Disease-associated Colorectal Neoplasia with Use of Thiopurines: A Systematic Review and Metaanalysis. J. Crohn’s Colitis.

[15] Kim E.R., Chang D.K. (2014). Colorectal cancer in inflammatory bowel disease: The risk, pathogenesis, prevention and diagnosis. World J. Gastroenterol..

[16] Beaugerie L., Svrcek M., Seksik P., Bouvier A., Simon T., Allez M., Brixi H., Gornet J., Altwegg R., Beau P. (2013). Risk of colorectal high-grade dysplasia and cancer in a prospective observational cohort of patients with inflammatory bowel disease. Gastroenterology.

[17] Kucharzik T., Dignass A.U., Atreya R., Bokemeyer B., Esters P., Herrlinger K., Kannengießer K., Kienle P., Langhorst J., Lügering A. (2020). Aktualisierte S3-Leitlinie Colitis ulcerosa—Living Guideline. Z. Für Gastroenterologie.

[18] Dyson J.K., Rutter M.D. (2012). Colorectal cancer in inflammatory bowel disease: What is the real magnitude of the risk?. World J Gastroenterol..

[19] Pinczowski D., Ekbom A., Baron J., Yuen J., Adami H.-O. (1994). Risk factors for colorectal cancer in patients with ulcerative colitis: A case-control study. Gastroenterology.

[20] Jackson S.S., Marks M.A., Katki H.A., Cook M.B., Hyun N., Freedman N.D., Kahle L.L., Castle P.E., Graubard B.I., Chaturvedi A.K. (2022). Sex disparities in the incidence of 21 cancer types: Quantification of the contribution of risk factors. Cancer.

[21] Jung Y.S., Han M., Park S., Kim W.H., Cheon J.H. (2017). Cancer Risk in the Early Stages of Inflammatory Bowel Disease in Korean Patients: A Nationwide Population-based Study. J. Crohn’s Colitis.

[22] Annese V., Beaugerie L., Egan L., Biancone L., Bolling C., Brandts C., Dierickx D., Dummer R., Fiorino G., Gornet J.M. (2015). European Evidence-based Consensus: Inflammatory Bowel Disease and Malignancies. J. Crohn’s Colitis.

[23] Axelrad J., Olen O., Sachs M., Erichsen R., Pedersen L., Halfvarson J., Askling J., Ekbom A., Sørensen H.T., Ludvigsson J. (2020). Inflammatory bowel disease and risk of small bowel cancer: A binational population-based cohort study from Denmark and Sweden. J. Crohn’s Colitis.

[24] Fields A.C., Hu F.Y., Lu P., Irani J., Bleday R., Goldberg J.E., Melnitchouk N. (2020). Small Bowel Adenocarcinoma: Is There a Difference in Survival for Crohn’s Versus Sporadic Cases?. J. Crohn’s Colitis.

[25] Kannengiesser K., Torsten K. (2018). Begleiterkrankungen bei CED Onkologische Fragen CED und Krebserkrankungen. Bauchredner.

[26] Preiß J.C., Bokemeyer B., Buhr H.J., Dignaß A., Häuser W., Hartmann F., Herrlinger K.R., Kaltz B., Kienle P., Kruis W. (2014). Aktualisierte S3-Leitlinie—”Diagnostik und Therapie des Morbus Crohn” 2014. Z. Für Gastroenterol..

[27] Tassone D., Ding N. (2020). A review of adverse events associated with immunosuppressive treatments in inflammatory bowel disease patients. J. Crohn’s Colitis.

[28] A-Rahim Y.I., Farrell R.J. (2019). Overview of Azathioprine and Mercaptopurine Use in Inflammatory Bowel Disease. http://112.2.34.14:9095/contents/overview-of-azathioprine-and-mercaptopurine-use-in-inflammatory-bowel-disease.

[29] Muller M., D’Amico F., Bonovas S., Danese S., Peyrin-Biroulet L. (2020). TNF inhibitors and risk of malignancy in patients with inflammatory bowel diseases: A systematic review. J. Crohn’s Colitis.

[30] Hashash J.A., Regueito M. (2019). Medical Management of Low-Risk Adult Patients with Mild to Moderate Ulcerative Colitis. https://www.uptodate.com/contents/medical-management-of-low-risk-adult-patients-with-mild-to-moderate-ulcerative-colitis.

[31] Sturm A., Atreya R., Bettenworth D., Bokemeyer B., Dignaß A., Ehehalt R., Germer C., Grunert P.C., Helwig U., Herrlinger K. (2022). Aktualisierte S3-Leitlinie “Diagnostik und Therapie des Morbus Crohn” der Deutschen Gesellschaft für Gastroenterologie, Verdauungs- und Stoffwechselkrankheiten (DGVS)-August 2021-AWMF-Registernummer: 021-004. Z. Für Gastroenterol..

[32] Bongartz T., Sutton A.J., Sweeting M.J., Buchan I., Matteson E.L., Montori V. (2006). Anti-TNF Antibody Therapy in rheumatoid arthritis and the risk of serious infections and malignancies: Systematic Review and Meta-analysis of rare harmful effects in randomized controlled trials. JAMA.

[33] Hyams J.S., Dubinsky M.C., Baldassano R.N., Colletti R.B., Cucchiara S., Escher J., Faubion W., Fell J., Gold B.D., Griffiths A. (2017). Infliximab Is Not Associated With Increased Risk of Malignancy or Hemophagocytic Lymphohistiocytosis in Pediatric Patients With Inflammatory Bowel Disease. Gastroenterology.

[34] Kucharzik T., Koletzko S., Kannengießer K., Dignaß A. (2020). Colitis ulcerosa—Diagnostische und therapeutische Algorithmen. Dtsch. Aerzteblatt Online.

