# Supplementary material for: Malignancy and Inflammatory Bowel Disease (IBD): Incidence and Prevalence of Malignancy in Correlation to IBD Therapy and Disease Activity—A Retrospective Cohort Analysis over 5 Years
Source: Biomedicines. 2025 Jun 6;13(6):1395. doi: 10.3390/biomedicines13061395 (PMC12189862; doi:10.3390/biomedicines13061395)
Supplement: Supplementary file 1 [file biomedicines-13-01395-s001.zip › biomedicines-3600782-supplementary.pdf]

Table S1. ages (at the time of the study, at the IBD diagnosis) and IBD duration of study patients.

	Age at the time of the study AM; median, (SD) [years]	Age at the diagnosis of IBD AM; median, (SD) [years]	IBD duration AM; median (SD) [years]
study cohort	46.83; 47.00 (15.64)	31.55; 28.00 (14.15)	15.13; 13.00 (10.82)
- male	46.79; 45.00 (16.43)	32.13; 28.00 (15.08)	14.55; 13.00 (10.11)
- female	46.86; 48.00 (14.53)	30.98; 28.00 (12.98)	15.70; 14.50 (11.20)
CD	47.54; 48.00 (14.61)	31.01; 27.00 (13.70)	16.45; 15.00 (11.45)
- male	46.88; 45.00 (15.26)	30.47; 26.50 (14.51)	16.23; 15.00 (10.20)
- female	48.12; 49.00 (14.44)	31.47; 28.00 (13.39)	16.65; 16.00 (12.30)
CU	45.61; 45.00 (17.41)	32.74; 28.00 (14.61)	12.58; 12.00 (9.48)
- male	47.54; 47.00 (18.42)	35.56; 30.00 (16.01)	11.98; 10.00 (9.65)
- female	43.34; 39.50 (16.39)	29.35; 28.00 (11.44)	13.31; 9.00 (9.78)
IBDU	45.50; 46.50 (16.00)	30.43; 27.00 (14.62)	15.07; 12.50 (8.70)
- male	40.13; 38.00 (14.29)	26.88; 27.00 (10.63)	13.25; 12.50 (8.75)
- female	52.67; 57.50 (16.50)	35.17; 37.50 (16.79)	17.50; 14.00 (8.80)

Table S2. age (at the time of the study, at the IBD diagnosis) and IBD duration of cancer patients.

	Age at the time of the study AM; median, (SD) [years]	Age at the diagnosis of IBD AM; median, (SD) [years]	IBD duration AM; median (SD) [years]
cancer patients with IBD	57.88; 60.00 (14.98)	40.00; 36.50 (17.69)	17.03; 14.00 (11.71)
- male	58.62; 63.00 (15.54)	41.00; 36.50 (19.08)	17.35; 14.00 (11.70)
- female	56.45; 58.00 (14.44)	38.00; 37.50 (16.36)	16.40; 13.00 (14.34)
CD	55.25; 58.50 (12.88)	36.68; 36.00 (14.81)	18.11; 14.00 (12.27)
- male	55.55; 59.00 (12.46)	35.30; 32.00 (12.89)	19.40; 15.50 (10.38)
- female	54.89; 58.00 (15.21)	38.22; 39.00 (17.34)	16.67; 12.00 (12.29)
UC	63.55; 68.00 (18.55)	46.80; 45.50 (22.96)	15.40; 14.00 (12.20)
- male	63.56; 68.00 (19.61)	48.00; 54.00 (24.02)	15.56; 14.00 (12.93)
- female	63.50; 63.50 (19.09)	36.00; 36.00 (-)	14.00; 14.00 (-)
IBDU	48.00; - (-)	35.00; - (-)	13.00; - (-)
- male	48.00; - (-)	35.00; - (-)	13.00; - (-)
- female	-	-	-

IBD and cancer. There were five patients who were first diagnosed with cancer followed by the diagnosis of IBD, it ranged from 3 to 7 years (AM 4.2 years, median 3 years).

Table S3: age at the diagnosis of cancer

	**Age at the diagnosis of cancer mean age; median (SD) [years]**
cancer patients with IBD	48,34; 48,00 (14,56)
- male	48,53; 48,00 (14,28)
- female	48,00; 50,50 (15,86)
CD	46,79; 50,00 (12,77)
- male	47,91; 51,00 (10,39)
- female	45,25; 48,00 (15,67)
UC	52,44; 48,00 (17,59)
- male	50,57; 47,00 (21,41)
- female	59,00; 59,00 (15,56)
IBDU	41,00; - (-)
- male	41,00; - (-)
- female	-

Table S4. characteristics of cancer patients with IBD considering the main groups of cancer, sex, age at the IBD diagnosis, age at the cancer diagnosis, year and age at the death. *1) Patient with rectal and thyroid cancer. *2) Patient with NET in term. Patients who first had IBD and then cancer were additionally marked in the table.

**main group**	**cancer type**	**sex**	**age [years]**	**IBD subtype**	**age at the IBD diagnosis [years]**	**age at the cancer diagnosis [years]**	**year of death; age at death**
skin cancer	SCC	**female***	**54**	**CD**	**12**	**43; 50**	**-**
		**female**	**67**	**CD**	**61**	**64**	**-**
		male	68	UC	54	unknown	-
	melanoma	**male**	**53**	**UC**	**37**	**38**	**-**
		**female**	**58**	**CD**	**57**	**58**	**-**
		female	77	UC	unknown	70	-
	BCC	**male**	**39**	**CD**	**25**	**32**	**-**
		male	86	UC	72	unknown	-
		female	61	CD	42	unknown	-
	highly differentiated verrucous carcinoma/BLT	**female**	**75**	**CD**	**39**	**59**	**-**
gastrointestinal cancer	colon cancer, NET of the appendix	female	34	CD	23	23	-
		**male**	**59**	**CD**	**42**	**56**	**-**
	colon cancer, coecum	male	69	CD	58	51	-
	colon cancer, transverse colon	male	47	CD	36	36	-
		**male**	**63**	**CD**	**28**	**60**	**-**
	colon cancer, descending colon	**male**	**39**	**CD**	**21**	**36**	**-**
	rectal cancer	**male*1)**	**65**	**UC**	**18**	**59**	**-**
		**female**	**50**	**UC**	**36**	**48**	**-**
	small intestine cancer, NET	male*2)	70	CD	48	48	-
hematologic diseases	AML	female	65	CD	56	53	-
		female	43	CD	31	26	-
	CML	**male*2)**	**70**	**CD**	**48**	**68**	**-**
	NHL	**male**	**38**	**CD**	**27**	**38**	**-**

	HL	**male**	**48**	**CI**	**35**	**41**	**-**
liver cancer	HCC	**male**	**58**	**CD**	**47**	**56**	**2019; 58 years**
	liver sarcoma	**male**	**49**	**UC**	**31**	**36**	**-**
prostate cancer	prostate cancer	male	75	UC	70	67	-
		**male**	**75**	**UC**	**60**	**70**	**-**
breast cancer	breast cancer	**female**	**37**	**CD**	**23**	**36**	**-**
renal cell carcinoma	renal cell carcinoma	male	79	UC	77	74	-
seminoma	seminoma	**male**	**22**	**UC**	**13**	**22**	**-**
thyroid cancer	thyroid cancer	**male*1)**	**65**	**UC**	**18**	**47**	**-**
CUP syndrome	CUP syndrome/ lung cancer	**male**	**65**	**CD**	**21**	**64**	**2014; 65 years**
otorhinolaryngological tumours	tongue cancer	male*3)	64	CD	unknown	50	-
	tonsil cancer	male*3)	64	CD	unknown	51	-

Table S5. characteristics of cancer patients with IBD considering the cancer diagnosis (incl. stage, the date of diagnosis), IBD, sex, year of the IBD diagnosis, cancer therapy and IBD therapy (before and after the cancer diagnosis). *1) Patient with rectal and thyroid cancer; *2) Patient with NET in terminal ileum and CML; *3) Patient with tongue and tonsil cancer. OP – operation, CTX – chemotherapy, ABT – antibody therapy, IT – immune therapy, RT – radiotherapy, AZA – azathioprine, ADA – adalimumab, CycA - cyclosporine A, GOLI – golimumab, INF – infliximab, MP – mercaptopurin, MTX - methotrexate, USTE – ustekinumab, VEDO – vedolizumab.

**cancer type**	**stage**	**sex**	**year of the IBD diagnosis**	**IBD**	**date of the cancer diagnosis**	**IBD therapy**		**cancer therapy**					**IBD therapy at the time of cancer diagnosis**		**IBD therapy after the cancer diagnosis**	
						drug	duration	OP	CTX	ABT	IT	RT	drug	duration	drug	duration
SCC		**female***	**1977**	**CD**	**08.2008**	**AZA AZA**	**1996 2000-unknown**	**X**					**-**	**-**	**unknown**	**unknown**
		**female**	**2013**	**CD**	**12.2016**	**-**	**-**	**X**					**AZA**	**01.2016–08.2017**	**ADA USTE**	**2017-2019 2019-further**
		male	2005	UC	unknown	AZA MTX INF VEDO	2010–2015 2016–2017 2017–2018 2018–further						unknown	unknown		
melanoma		**male**	**2003**	**UC**	**2004**	**-**	**-**	**X**					**CycA**	**2004**	**AZA**	**2005**
		**female**	**2018**	**CD**	**07.2019**	**-**	**-**	**X**					**USTE**	**03.2019**	**USTE**	**further**
	pT1a N0 M0 S0	female	unknown	UC	07.2012	-	-	X					-	-	AZA	2016
BCC		**male**	**2005**	**CD**	**2012**	**AZA**	**2005–unknown**	**X**					**AZA**	**2005–unknown**	**ADA**	**2016-further**
		male	2005	UC	unknown	AZA MP	2007 2007–further						unknown		MP	further
		female	2000	CD	unknown	AZA	2013						unknown		-	-

**highly differentiated verrucous carcinoma/BLT**		**female**	**1983**	**CD**	**2003**	**AZA**	**2000**	**X**				**-**	**-**	**-**	**-**
NET of the appendix		female	2008	CD	08.2008	-	-	X				-	-	AZA<br>MP	2009-2012<br>2013-2015
	**pT1a pN0 R0**	**male**	**2002**	**CD**	**05.2016**	**-**	**-**	**X**				**-**	**-**	**AZA**<br><br>**ADA**<br>**USTE**	**12.2016**<br>**01.2017**<br>**2017-2018**<br>**2018-further**
coecum cancer	pT3 pN2 cM0 G2	male	2008	CD	03.2001	-	-	X	X			-	-	-	-
colon cancer, transverse colon	pT3 N1 Mx	male	2008	CD	11.2008	-	-	X	X			-	-	AZA<br>INF<br>ADA<br>VEDO<br>USTE<br>MTX	2010-2019<br>2013<br>2014<br>2014-2016<br>2017<br>2018
	**pT3 pN0 L0 V0 Pn0 R0**	**male**	**1984**	**CD**	**12.2016**	**-**	**-**	**X**				**-**	**-**	**-**	**-**
**colon cancer, descending colon**	**pT2 pN0 L0 V0 R0 G2**	**male**	**2001**	**CD**	**01.2016**	**INF**	**2011–2012**	**X**				**AZA**	**2015–2016**	**ADA**	**2019-further**
rectal cancer	**uT3 cN0 cM0**	**male[*1)]**	**1972**	**UC**	**04.2013**	**AZA**	**2008–2009**		**X**		**X**	**-**	**-**	**-**	**-**
	**pT2 pN0 L0 V0 R0 G1**	**female**	**2005**	**UC**	**04.2017**	**AZA**	**2007–2010**	**X**				**-**	**-**	**-**	**-**
small intestine cancer, NET		male[*2)]	1997	CD	10.1997	-	-	X				-	-	AZA<br>ADA<br>MTX<br>INF<br>USTE<br>VEDO	1999-2002<br>2012-2013<br>2016-2017<br>09-10.2017<br>2017-2018<br>2018-further
AML		female	2010	CD	10.2007	-	-		X		X	-	-	AZA<br>MTX<br>VEDO	2010-2015<br>2016-2019<br>2019
		female	2007	CD	12.2002	-	-		X			-	-	AZA<br>ADA<br>INF	2010-2012<br>2012-2017<br>2018-further
CML		**male[*2)]**	**1997**	**CD**	**04.2017**	**AZA**<br>**ADA**	**1999–2002**<br>**2012–2013**			**X**		**MTX**	**2016–2017**	**INF**<br>**USTE**<br>**VEDO**	**09-10.2017**<br>**2017-2018**<br>**2018-further**
NHL	II E B	**male**	**2008**	**CD**	**03.2019**	**AZA**	**2015–2018**		**X**		**X**	**ADA**	**2018–2019**	**-**	**-**

HL	CS IVB	male	2006	CI	12.2012	AZA	2007-2012		X			AZA<br>INF	2007–2012<br>2012	-	-
HCC	T3b Nx M0	male	2008	CD	2017	AZA	2012				X	-	-	-	-
liver sarcoma	Stadium IV	male	2001	UC	07.2006	-	-	X	X			-	-	-	-
prostate cancer	5/10 Gleason	male	2014	UC	04.2011	-	-				X	-	-	MTX<br>MP<br>GOLI<br>INF<br>VEDO	2015<br>2016-2017<br>2016<br>2017<br>2017-2018
	pT2a, pN0, Pn1, R1	male	2004	UC	06.2014	AZA	2008	X			X	-	-	MP<br>INF<br>VEDO	2015<br>2015-2016<br>2017-further
breast cancer	pTis N0, G3	female	2005	CD	2018	-	-	X	X	X		AZA	2008–further	AZA	2008-further
renal cell carcinoma	pT1a pNx L0 V0 G2 R0	male	2017	UC	11.2014	-	-	X				-	-	-	-
seminoma	pT2 cN0 L0 V1 S1 R0	male	2010	UC	06.2019	-	-	X	X			-	-	-	-
thyroid cancer	pT1b N0 M0	male*1)	1972	UC	2001	-	-	X				-	-	AZA	2008-2009
CUP-syndrome/ lung cancer	cT4 cN0 cM0	male	1970	CD	07.2013	AZA	1999–2006		X		X	-	-	-	-
tongue cancer		male*3)	unknown	CD	12.2005	AZA	n. b.	X				AZA	unknown	AZA	2018-further
tonsil cancer	T1 N0 M0	male*3)	unknown	CD	08.2006	AZA	n. b.	X				AZA	unknown	AZA	2018-further
